# Mind Perception: Real but Not Artificial Faces Sustain Neural Activity beyond the N170/VPP

**DOI:** 10.1371/journal.pone.0017960

**Published:** 2011-03-31

**Authors:** Thalia Wheatley, Anna Weinberg, Christine Looser, Tim Moran, Greg Hajcak

**Affiliations:** 1 Department of Psychological and Brain Sciences, Dartmouth College, Hanover, New Hampshire, United States of America; 2 Department of Psychology, Stony Brook University, Stony Brook, New York, United States of America; Dalhousie University, Canada

## Abstract

Faces are visual objects that hold special significance as the icons of other minds. Previous researchers using event-related potentials (ERPs) have found that faces are uniquely associated with an increased N170/vertex positive potential (VPP) and a more sustained frontal positivity. Here, we examined the processing of faces as objects vs. faces as cues to minds by contrasting images of faces possessing minds (human faces), faces lacking minds (doll faces), and non-face objects (i.e., clocks). Although both doll and human faces were associated with an increased N170/VPP from 175–200 ms following stimulus onset, only human faces were associated with a sustained positivity beyond 400 ms. Our data suggest that the N170/VPP reflects the object-based processing of faces, whether of dolls or humans; on the other hand, the later positivity appears to uniquely index the processing of human faces—which are more salient and convey information about identity and the presence of other minds.

## Introduction

Faces are the observable icons of unobservable minds. People read faces, and the eyes in particular, for cues to emotion, intention, and social meaning [1]. Although indications of animacy can be gleaned from other cues at greater, and safer, distances, faces are uniquely suited to convey information about other minds. As such, they are among the most important objects in the visual environment—faces capture our attention, and orient us to other minds that can think, feel, and interact with our own. This preferential attention to faces is present from birth, suggesting that some aspects of face processing are innate. Newborns are born with primitive face “detectors” that help them orient to their caregivers thereby aiding survival [2]. Preferential attention to faces does not end with infancy; faces continue to capture attention as development progresses from physical dependency to social awareness. By age four, children attend to faces as powerful icons of other minds [3].

The lifelong importance of face detection, which offers cues to mind distinct from other cues to animacy, is reflected in its privileged processing in the brain. It is now well-established that a region along the lateral fusiform gyrus responds more to faces than to other objects or scrambled faces [4], [ 5], [ and 6]. Faces are also associated with a specific and rapid electrocortical signature: research using event related potentials (ERPs) suggests that faces, but not other objects, evoke a distinct brain potential with a peak latency around 170 ms [7], [ 8], [ 9], [ 10], [ and 11]. This component manifests as a negative-going potential at bilateral occipital-temporal sites. It is referred to as the N170 when an average of all electrodes is used as a reference and is a observed as a centrally distributed positive-going potential called the vertex positive potential (VPP) when an average of mastoid electrodes is used as a reference [12].

This N170/VPP is evoked by all types of faces, including schematic line drawings of faces [7], [ 9], [ 10], [ and 11]. This broad response profile suggests a rapid pattern-matching mechanism [13], [ 14], [ and 15] that flags input as a potential face.

Such a pattern-matching process is inherently prone to making errors—false alarms in particular, because many inanimate objects can appear superficially face-like. Indeed, it is common to see a face in clouds, house facades, or the front grills of cars. Having a rapid, but error-prone, first stage of detection is consistent with the tenets of signal detection theory, which posits that a liberal criterion should apply whenever the cost of a missed stimulus is higher than the cost of a false alarm [16]. This is the same principle used in the design of smoke alarms, which are intentionally sensitive enough to err on the side of ‘detecting smoke’ even in cases where there is none. A conservative smoke alarm that activated only after a high threshold had been passed would be slow and prone to incorrect rejections. The potentially fatal consequences of incorrect rejections (failing to report smoke when smoke is really present) are obvious in this case. In the domain of faces, a false alarm such as occurs when we see faces in clouds, house facades, or car grills is an acceptable tradeoff for the ability to rapidly detect a potential friend or foe [17].

Although a rapid and liberal face detection mechanism makes sense in the service of survival, we must also have some way to discount false alarms. We must be able to discriminate faces worthy of our thoughts, feelings, and actions from false alarms that are not actually faces. Otherwise we might regard clouds, cars, or houses as objects with a mental life. The fact that we typically do not interact with line drawings or ponder the mental lives of dolls suggests that this discrimination occurs obligatorily. In short, detecting real human faces may require a two-stage process: (1) the rapid, liberal detection of a face pattern followed by (2) the evaluation of that face for its relevance as a cue to another mind. Following the N170/VPP, salient faces (e.g., familiar faces, faces expressing emotion) elicit a sustained positive ERP relative to less salient faces (unfamiliar faces, neutral expressions [18], [ 19], [ 20], [ 21], [ 22], [ 23], [ and 24]). This positive potential may index an allocation of mental resources, such as attention, based on the biological relevance of the face being viewed. This resource allocation would be consistent with the finding that emotional faces are better remembered than neutral faces [23]. The frontal positivity elicited by faces appears similar to the late positive potential (LPP)—a sustained positivity in the ERP following emotional compared to neutral stimuli [25], [ 26], [ and 27]. Thus, it is possible that the frontal positivity elicited by faces may index a process of elaboration and encoding of actual faces beyond the earlier and coarser N170/VPP.

Here we used images of human and doll faces in order to dissociate event-related potential (ERP) components that index the recognition of faces as specific visual objects vs. indicators of other minds. Participants viewed photographs of human faces, doll faces, and clocks as ERPs were recorded. Both kinds of faces were predicted to elicit an equivalent early response related to face perception (i.e., the VPP) relative to the perception of non-face objects (i.e., clocks). In addition, we predicted that the ERP elicited by human and doll faces would diverge at longer latencies. Based on existing data linking later midline positive potentials to motivationally salient emotional stimuli [25], [ 28], [ and 29], including more salient faces [20] [ and 23], we predicted that human faces would be uniquely characterized by a more sustained, late positive potential over frontal and central regions.

## Methods

### Participants

A total of 19 Stony Brook University undergraduates (7 female) participated in the study for course credit. The average age was 18.82 years (*sd* = .81); 58% of the sample was Caucasian, 11% was African-American, 16% was Asian or Asian-American, and 5% was Hispanic. All participants provided written consents and procedures were approved by the Institutional Review Board of Stony Brook University.

### Visual Stimuli

**Figure 1:**
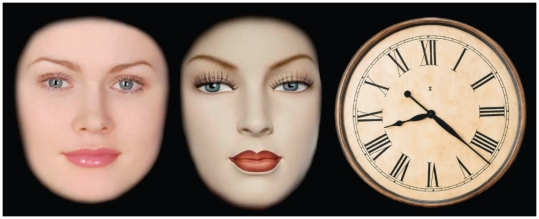
Stimulus exemplars from the three categories: Human Faces, Doll Faces, Clocks.

Sixty full color photographs were used as stimuli. Twenty depicted human faces, 20 depicted doll faces, and 20 were pictures of clocks. All stimuli were cropped to expose only the face or the entire clock and placed on a black background. The luminance of the stimuli was equated across categories. See figure 1 for an exemplar of each stimulus category. All visual stimuli were presented on a Pentium D computer, using Presentation software (Neurobehavioral Systems, Inc.; Albany, California). Prior to each trial, participants viewed a white fixation cross on a black background. Each picture was displayed in color at the full size of the monitor, 48.26 cm. Participants were seated approximately 70 cm from the screen and the images occupied about 40° of visual angle horizontally and vertically.

### Procedure

Participants were given verbal instructions indicating that they would passively view various pictures. Once seated, electroencephalograph sensors were attached. On each trial, a picture was presented for 1,000 ms, followed by a variable inter-trial interval consisting of a blank screen, ranging from 1,500 ms to 1,900 ms. During the experiment, each picture was presented once in random order. Once all pictures were presented, the pictures were presented a second time, again in a randomized order. Pictures were repeated twice to increase signal-to-noise ratio in the ERPs.

### Electroencephalographic Recording and Data Processing

Continuous EEG was recorded using a custom cap (Cortech Solutions, Wilmington, N.C., USA) and the ActiveTwoBioSemi system (BioSemi, Amsterdam, Netherlands). The signal was preamplified at the electrode with a gain of 16x; the EEG was digitized at 64-bit resolution with a sampling rate of 512 Hz using a low-pass fifth-order sinc filter with a half-power cutoff of 102.4 Hz. Recordings were taken from 64 scalp electrodes based on the 10/20 system, as well as two electrodes placed on the left and right mastoids. The electrooculogram was recorded from four facial electrodes placed 1 cm above and below the left eye, 1 cm to the left of the left eye, and 1 cm to the right of the right eye. Each electrode was measured online with respect to a common mode sense electrode that formed a monopolar channel. Off-line analysis was performed using Brain Vision Analyzer software (Brain Products, Munich, Germany). All data were re-referenced to the average of all scalp electrodes and band-pass filtered with cutoffs of 0.1 and 30 Hz. The EEG was segmented for each trial, beginning 200 ms before picture onset and continuing for 1,200 ms (i.e., the entire picture presentation duration). Each trial was corrected for blinks and eye movements using the method developed by Gratton and colleagues [30]. Specific channels were rejected in each trial using a semi-automated procedure, with physiological artifacts identified by the following criteria: a step of more than 50 µV between sample points, a difference of 300 µV within a trial, and a maximum difference of less than 0.5 µV within 100-ms intervals. Additional physiological artifacts were visually identified and removed from further analysis.

Stimulus-locked ERPs were averaged separately for human faces, doll faces, and clocks. The vertex positivity (VPP) was scored as the average activity in a 175–200 ms window at Cz where the vertex positivity was largest for both human and doll faces. To evaluate the later positive component, we similarly examined the average activity in a 400–1000 ms window following picture presentation. The difference between facial stimuli (i.e., both doll and human faces) and clock stimuli was maximal at Cz, whereas the difference between human and doll faces was maximal at AFz. The late positivity was analyzed at AFz, although the statistical analyses were identical at Cz.

In order to evaluate the VPP and the later positivity, repeated measures ANOVAs were conducted using SPSS (Version 15.0) General Linear Model software, with Greenhouse-Geisser correction applied to *p*-values associated with multiple-df, repeated measures comparisons when necessitated by violation of the assumption of sphericity. When appropriate, post-hoc comparisons were conducted using paired-samples t-tests; *p*-values were adjusted as noted with the Bonferroni correction for multiple post-hoc comparisons.

## Results

### Vertex Positivity

**Figure 2:**
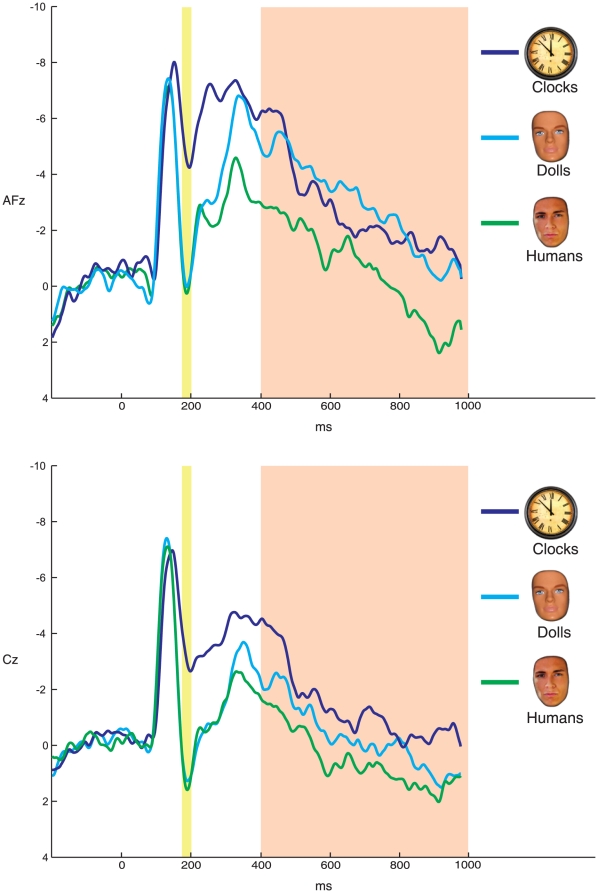
Stimulus-locked ERPs. ERPs elicited by human faces, doll faces, and clocks at frontal and central recording sites AFz (top) and Cz (bottom), respectively. The vertex positivity is highlighted in the yellow shaded region. The VPP is evident as a positive deflection maximal around 180 ms and is larger (i.e., more positive) for both human and doll faces relative to clocks. This difference is maximal at Cz (bottom graph). However, human faces elicited a larger later positive potential relative to both clocks and doll faces. This difference began following the vertex positivity and continued for the duration of stimulus presentation (highlighted in the orange shaded region). The LPP was maximal at AFz (top).

**Figure 3:**
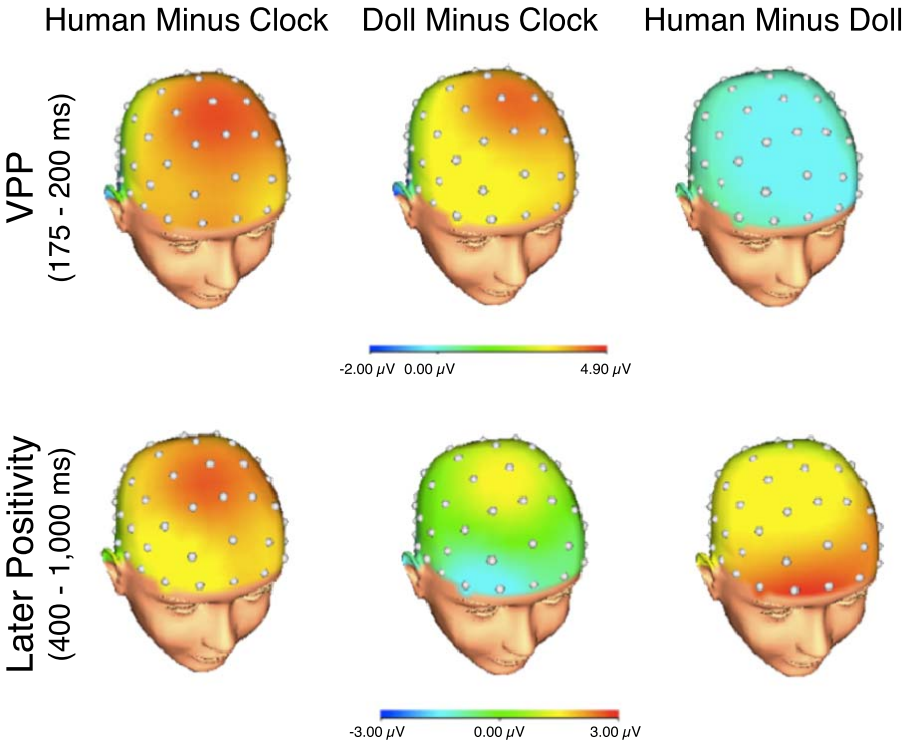
Scalp distributions of ERP differences. Scalp distributions of the difference between human faces and clocks (left), doll faces and clocks (middle), and human faces and doll faces (right) in the time range of the vertex positivity (i.e., 175–200 ms; top) and later positivity (i.e., 400–1,000 ms; bottom). Relative to clocks, both human and doll faces elicited an increased vertex positivity (top). However, human faces elicited an increased later positivity relative to both clocks and doll faces (bottom).

No ERP differences were evident between the first and second presentations of the faces, and thus, the remainder of the paper collapses across presentations. Grand average stimulus-locked ERPs elicited by images of clocks, doll faces, and human faces, are presented in Figure 2 at two midline frontal-central sites: AFz (top) and Cz (bottom). Figure 3 presents topographic maps depicting voltage differences (in µV) for human faces minus clocks (left), doll faces minus clocks (center) and human faces minus doll faces (right) in the time-range of the VPP (top) and later positivity (bottom).

Mean VPP area measures are presented in Table 1. As indicated in Figures 2 and 3, the VPP peaked between 175 and 200 ms and was maximal over Cz. Confirming the impressions from Figures 2 and 3, the VPP varied significantly as a function of picture type (*F*(2,36) = 50.94, *p*<.001, η_p_ *^2^* = 0.74), such that both human faces (*M* = −0.51 *sd* = 2.43) and doll faces (*M* = −0.59, *sd* = 3.03) elicited a significantly more positive response than clocks (*M* = −4.33, *sd* = 3.17; *t*(18) = 8.36, p<.001 and *t*(18) = 9.10, *p*<.001, respectively). However, the VPP was equivalent in magnitude for doll and human faces (*t*(18) = .17, *p*>.85; critical *p*-value = .02 for three comparisons), suggesting the VPP was uniquely sensitive to faces, but did not differentiate between doll and human faces.

**Table 1:** Mean ERP area measures (µV) for the VPP and the frontal positivity when viewing different stimulus types (*SD*s in parentheses).

Picture Type	Vertex Positivity	Later Positivity
Clocks	−4.34 (3.17)	−2.57 (5.78)
Doll Faces	−.59 (3.03)*	−2.76 (4.93)
Human Faces	−.51 (2.43)*	−.44 (5.86)* †

*Note:*

* indicates p<.01 when compared to clocks,

*† indicates p<.01 when compared to doll faces.*

The VPP has been shown to be the positive end of the same dipole as the N170 [12]. Whether the VPP or the N170 is observed depends on whether a mastoid or average reference is used. When referenced to the average reference, the O1 and O2 electrodes in this study showed a maximal N170 response consistent with previous research. Data were scored in the same time-window as the VPP (175–200 ms), at the average of these occipital electrodes. Like the VPP, the N170 varied as a function of picture type (*F*(2,36) = 8.47, *p*<.001, η_p_ *^2^* = 0.32), such that both human faces (*M* = 3.32 *sd* = 2.26) and doll faces (*M* = 4.39, *sd* = 3.96) elicited a more negative response than clocks (*M* = 6.86, *sd* = 4.39; *t*(18) = 3.39, p<.01 and *t*(18) = 4.29, *p*<.001, respectively). The N170 was also equivalent in magnitude for doll and human faces (*t*(18) = 1.12, *p*>.05; critical *p*-value = .02 for three comparisons). Thus, the N170 (using the average reference) and the VPP (using a mastoid reference) yielded identical results: both components were uniquely sensitive to faces, but did not differentiate between doll and human faces.

### Later Positivity

 As indicated in Figures 2 and 3, human faces were uniquely characterized by a later positivity, which was maximal at AFz. This later positivity became larger by approximately 400 ms following the presentation of human faces relative to both doll faces and clocks—and this difference was sustained for the duration of picture presentation. At AFz, the later positivity varied as a function of picture type (*F*(2,36) = 5.65, p<.05, η_p_ *^2^* = .24), such that human faces (*M* = −.44 *sd* = 5.86) elicited a significantly more positive response than clocks (*M* = −2.57, *sd* = 5.78; *t*(18) = 3.70, *p*<.005) and doll faces (*M* = −2.76, *sd* = 4.93; *t*(18) = 3.13, *p*<.01). However, doll faces did not elicit a significantly different response from clocks (*t*(18) = .20, *p*>.80; critical *p*-value = .02 for three comparisons), suggesting that the later positivity uniquely distinguished human faces.

 Because differences between doll faces and clocks, and human faces and clocks, appeared maximal at Cz (Figure 2, bottom left and right), analyses were also conducted at this site to examine differences between stimulus types. Consistent with the results at AFz, the later positivity at Cz varied significantly as a function of picture type (*F*(2,36) = 12.80, p<.001, η_p_ *^2^* = .42), such that human faces (*M* = −.20 *sd* = 4.09) elicited a significantly larger (more positive) response than doll faces (*M* = −1.92, *sd* = 2.78; t(18) = 2.87, p<.01), and clocks (*M* = −3.12, *sd* = 4.03); t(18) = 5.15, p<.001). Also, doll faces did not elicit a significantly different response at Cz compared to clocks (t(18) = 2.09, p>.05;critical *p*-value = .02 for three comparisons).

## Discussion

Previous researchers have suggested that the visual system has a general perceptual architecture that supports rapid, parallel, and feed-forward processes in the service of survival (“vision at a glance” [31] [ and 32]), particularly for face detection [13] and slower, more detailed analyses involving iterative frontal-temporal feedback in the service of meaning (“vision for scrutiny” [31] [ and 33]). Consistent with this view and other findings in the literature [7], [ 9], [ 10], [ 11], [ 34], [ and 35], we find that both human and doll faces are associated with an early electrophysiological response (N170/VPP) relative to non-face objects. However, here we show that only human faces sustain activity beyond the N170/VPP, in the form of a later positive potential (∼400 ms post-stimulus). Previous researchers have suggested that later positivities, such as the one reported here, are sensitive to the salience and meaning of visual stimuli. In particular, frontal and central positivities in this time range are enhanced for stimuli more directly related to biological imperatives [25]. Likewise, explicit manipulations of the meaning of a stimulus influence the magnitude of these later positivities [26], [ 28], [ 29], [ and 36]. Together, these findings suggest that face perception employs at least two processing stages, one in which faces are rapidly detected and another in which faces are processed for their potential relevance as an emblem of another mind. The lack of an explicit task in the present experiment suggests that both of these processes unfold automatically when viewing faces, albeit at different timescales.

That these processing stages unfold automatically just by viewing a face suggests that the perception of mind may be an obligatory perceptual inference consistent with Helmholtz's “unconscious inferences” [37]. For example, just as people cannot help but “see” material and pigment given particular patterns of color and luminance, people cannot help but “see” mind in a face given a particular pattern of visual cues. Thus, it would be appropriate to distinguish “social perception,” which results from rapid, automatic, and unconscious inferences about other minds on the basis of cues such as facial expressions (e.g. “He is angry”), from “social cognition,” which results from inferences about other minds based on the outputs of the first stage of social perception (e.g. “He must be angry because I cancelled our dinner plans”). Social cognition therefore encompasses modeling the contents of another's mind (“theory of mind”) once a mind has been perceived. The present study examines the information processing architecture underlying visual social perception.

Although face detection is associated with the earlier of the two potentials observed here, we do not claim that face detection is a necessary first step for the perception of mind. People can discern the presence of a mind when viewing only the eye region of a face [1] [ and 38]. People also automatically attribute minds to simple geometric shapes that move in non-Newtonian ways (e.g., self propulsion and interactivity [39], [ 40], [ 41], [ 42], [ 43], [ and 44]. The present study, however, only concerns face perception. We suggest that visual input is first matched to a face-pattern template. Once a face is detected, it is subsequently evaluated for its relevance to the perceiver. As none of the faces in the present study held any particular relevance for the participants (e.g., personally familiar, emotionally expressive), the observed later positivity to human faces may index the perception of visual cues to mind. This later positivity for human faces could also reflect generic attentional, affective, and memory processes engaged by human faces relative to doll faces. That is, human faces may be more interesting, affecting, and memorable than doll faces. We suggest that increased domain-general processing for human faces is likely rooted in the perception of mind; e.g., cues to mind signal that a face is worthy of continued monitoring. However, it is also possible that the later positivity indexes domain-general processing wholly independent of mind perception that nonetheless is greater for human faces relative to doll faces (e.g., human faces may be more familiar than doll faces and thus more rewarding). Finally, it is also possible that participants adopted category-specific processing strategies unrelated to mind perception to make the task more interesting and that these differences were consistent across participants.

While the later positivity observed for human faces may index a perception of mind, it may also be characterized as indexing a perception of animacy. It is unclear whether these attributes can be dissociated in a face as they almost always co-occur in reality, but non-face body parts may help elucidate the matter. If the later positivity difference between human and doll faces is also present between human and doll hands, for example, animacy perception may be a more accurate characterization of the second process than mind perception. Finally, the later positivity for human faces observed here might occur for any stimuli that convey the presence of a mind, such as Heider and Simmel-type animations. The present results cannot determine whether the later positivity has characteristics peculiar to face perception. Future research will have to clarify these matters.

In sum, these data suggest that the processing cascade from early face perception to later mind perception can be indexed using specific ERP components. This two stage processing architecture may simultaneously allow for rapid detection followed by discounting false alarms in face perception. This may explain why we can immediately detect faces in our midst while reserving more intensive social-cognitive resources for only those faces that are actually capable of thinking, feeling, and interacting with us.
